# 

**Published:** 1845-10

**Authors:** 


### CONTENTS

### OF NO. IV.

### ORIGINAL COMMUNICATIONS.

### ESSAYS AND CASES.

ART. I.—Cases of Erysipelatous Laryngitis, or Black-Tongue. By A. M. KELLER, M.D., of Courtland, Ala. - - - 277

ART. II.—An Account of Epidemic Erysipelas, as it occurred at Columbia, Tenn. Read to the Medical Society of Tennessee, at its meeting in Nashville, May, 1845. By H. R. ROBARDS, M.D., of Columbia, Tenn. - - - - 285

ART. III.—A Case of Inflammation and Suppuration of the Colon, excited by the presence of a river pebble—its discharge after remaining in the bowels seven years, and recovery of the patient. Read to the Medical Society of Tennessee, May, 1845. By J. IRWIN, M.D. - - - - - - 297

ART. IV.—Two Cases of Parturition, with Remarks. Read before the Medical Society of Tennessee, at its Annual Meeting, in May, 1845. By JNO. W. RICHARDSON, M.D. of Rutherford county. - - - - - - - - 298

ART. V.—Case of a Gun-Shot Wound in the Back, followed by Hæmaturia, but no other alarming symptom. Read before the Medical Society of Tennessee at the Annual Meeting in May, 1845. By J. W. STOUT, M. D., of Nashville, Tenn. - - - - - - - - - - 304

ART. VI.—Case of Injury of the Leg resulting in Mortification and Death. By JESSE HARVEY, M.D., of Harveysburg, Ohio, - - - - - - - - . - 306

### REVIEWS.

ART. VII.—Vestiges of the Natural History of Creation. Second edition, from the third London edition, greatly amended by the author, and an iutroduction, by Rev. GEORGE B. CHEEVER, D.D. N. York: Wiley & Putnam. 1845. 12mo. pp. 280. - - - - - - - - 308

Art. VIII.—The Principles and Practice of Dental Surgery. By Chapin A. Harris, M. D., D. D. S., Professor of Practical Dentistry and Dental Pathology in the Baltimore College of Dental Surgery; Fellow of the American Society of Dental Surgeons; Member of the Medico-Chirurgical Faculty of Maryland, &c., &c. Second Edition: revised, modified, and greatly enlarged. Illustrated by 69 wood engravings. - - - - - - - - 319

SELECTIONS FROM AMERICAN AND FOREIGN JOURNALS

Case of Traumatic Tetanus successfully treated by large quantities of Wine and Brandy, with other means, - - - 325
Treatment of Neuralgia by Tobacco. - - - - - 326
Average of cases of Consumption Cured. - - - - - 328
Extract of Green Walnut Shells, in Chronic Congestion of the Tonsils. - - - - - - - - - - 328
Treatment of Infantile Gastric Fever. - - - - - 328
Urinary Deposits and Treatment. - - - - - - 329
On certain Differences of the Composition of Blood in the Male and Female. - - - - - - - - - 342
True and False Mesmerism. - - - - - - - 343
Paralysis of the Arm Relieved. - - - - - - 344
State of the Blood, produced by the Absorption of Urine. - - 345
How to Preserve Pills of the Extract of Aloes. - - - - 345
Use of Arsenic in Ascities. - - - - - - - 346
Luxations. - - - - - - - - - - 346
Case of Continued Priapism. - - - - - - - 350
Clinical Lectures on Medicine. - - - - - - 353
Secrets of Homœopathy—Hahnemann and his System. - - 359

ORIGINAL INTELLIGENCE.

Death of Professor Richardson. - - - - - - 361
Lightning Rods, - - - - - - - - - 364
Vacancy in Transylvania University. - - - - - 367
Rush Medical College. - - - - - - - - 367
The case of Dr. Baker—Murder—Monomania. - - - 368

